# Early prediction of neoadjuvant chemotherapy response by exploiting a transfer learning approach on breast DCE-MRIs

**DOI:** 10.1038/s41598-021-93592-z

**Published:** 2021-07-08

**Authors:** Maria Colomba Comes, Annarita Fanizzi, Samantha Bove, Vittorio Didonna, Sergio Diotaiuti, Daniele La Forgia, Agnese Latorre, Eugenio Martinelli, Arianna Mencattini, Annalisa Nardone, Angelo Virgilio Paradiso, Cosmo Maurizio Ressa, Pasquale Tamborra, Vito Lorusso, Raffaella Massafra

**Affiliations:** 1 Struttura Semplice Dipartimentale di Fisica Sanitaria, I.R.C.C.S. Istituto Tumori “Giovanni Paolo II”, Viale Orazio Flacco 65, 70124 Bari, Italy; 2 Dipartimento di Matematica, Università Degli Studi di Bari, 70121 Bari, Italy; 3 Struttura Semplice Dipartimentale di Chirurgia, I.R.C.C.S. Istituto Tumori “Giovanni Paolo II”, Viale Orazio Flacco 65, 70124 Bari, Italy; 4 Struttura Semplice Dipartimentale di Radiologia Senologica, I.R.C.C.S. Istituto Tumori “Giovanni Paolo II”, Viale Orazio Flacco 65, 70124 Bari, Italy; 5 Unità Operativa Complessa di Oncologia Medica, I.R.C.C.S. Istituto Tumori “Giovanni Paolo II”, Viale Orazio Flacco 65, 70124 Bari, Italy; 6 Interdisciplinary Center for Advanced Studies on Lab-on-Chip and Organ-on-Chip Applications (ICLOC), University of Rome Tor Vergata, 00133 Rome, Italy; 7 Dipartimento di Ingegneria Elettronica, Università di Roma Tor Vergata, Via del politecnico 1, 00133 Rome, Italy; 8 Unita Opertiva Complessa di Radioterapia, IRCCS Istituto Tumori ”Giovanni Paolo II”, 70124 Bari, Italy; 9 Oncologia Sperimentale e Biobanca, I.R.C.C.S. Istituto Tumori “Giovanni Paolo II”, Viale Orazio Flacco 65, 70124 Bari, Italy; 10 Unità Operativa Complessa di Chirurgica Plastica e Ricostruttiva, I.R.C.C.S. Istituto Tumori “Giovanni Paolo II”, Viale Orazio Flacco 65, 70124 Bari, Italy

**Keywords:** Medical imaging, Cancer, Breast cancer, Cancer therapy

## Abstract

The dynamic contrast-enhanced MR imaging plays a crucial role in evaluating the effectiveness of neoadjuvant chemotherapy (NAC) even since its early stage through the prediction of the final pathological complete response (pCR). In this study, we proposed a transfer learning approach to predict if a patient achieved pCR (pCR) or did not (non-pCR) by exploiting, separately or in combination, pre-treatment and early-treatment exams from I-SPY1 TRIAL public database. First, low-level features, i.e., related to local structure of the image, were automatically extracted by a pre-trained convolutional neural network (CNN) overcoming manual feature extraction. Next, an optimal set of most stable features was detected and then used to design an SVM classifier. A first subset of patients, called fine-tuning dataset (30 pCR; 78 non-pCR), was used to perform the optimal choice of features. A second subset not involved in the feature selection process was employed as an independent test (7 pCR; 19 non-pCR) to validate the model. By combining the optimal features extracted from both pre-treatment and early-treatment exams with some clinical features, i.e., ER, PgR, HER2 and molecular subtype, an accuracy of 91.4% and 92.3%, and an AUC value of 0.93 and 0.90, were returned on the fine-tuning dataset and the independent test, respectively. Overall, the low-level CNN features have an important role in the early evaluation of the NAC efficacy by predicting pCR. The proposed model represents a first effort towards the development of a clinical support tool for an early prediction of pCR to NAC.

## Introduction

Neoadjuvant chemotherapy (NAC) is commonly used as an initial treatment for locally advanced breast cancers or breast cancers with specific molecular histotypes^1,2^: monitoring of response to treatment could allow for breast-conserving surgery.

The efficacy of NAC is assessed through the clinical and radiological response by using the Response Evaluation Criteria In Solid Tumors (RECIST)^3,4^ during treatment and the pathological complete response after surgery. The pathological Complete Response (pCR) indicates the absence of residual invasive disease or metastatic lymph nodes at the end of the entire course of the therapy. The rates of pCR are significantly higher in Triple Negative and HER2+ tumors rather than in luminal tumors with positive estrogen and progesterone receptors (ER+/PgR+/HER2−)^2^, whereas there is a small portion of patients who do not respond to the therapy resulting in disease progression^5^.

The achievement of pCR may be considered as an independent predictor of better disease-free survival with the possibility to hypothesize a future non-need for surgery for patients who achieve pCR after NAC (responders)^3,4^. Therefore, an early identification of those patients who are responders or non-responders would be beneficial to improve and personalize the treatment planning thus sparing patients from potentially ineffective and/or toxic treatment. Indeed, early responder patients, i.e., responders even since the early stages of NAC, are more likely to take advantage from breast conserving surgery, avoiding them a full mastectomy^6^.

Within this emerging scenario, a systematic literature search has highlighted the Dynamic Contrast-Enhanced Magnetic Resonance Imaging (DCE-MRI) as an indispensable tool for monitoring the response to therapy^7–10^. For instance, changes in morphological and kinetic parameters extracted from DCE-MRI, as well as from diffusion weighted imaging, have been demonstrated to be helpful in predicting treatment response and pCR^11–14^. However, MRI has limitations when clinically used because influenced by inter-intra-observer variability^15^. For this reason, the radiomic analysis of DCE-MR images by means of automatic and semi-automatic computerized systems developed by experts has become of great interest as evidenced in various breast imaging methods^16–19^. Radiomic features, such as tissue, peritumoral or intratumoral features, extracted from raw images and appropriately combined with histological variables could help to predict the progress of the oncologic disease as well as pCR ever since the early stages of NAC^1,7,14,20–23^. In particular, the background parenchymal enhancement (BPE) parameter has been demonstrated to be a predictive factor of disease course and response to neoadjuvant therapy in breast cancer^24,25^.

Nevertheless, human expertise and a great effort in terms of feature engineering are required to convert images in valuable radiomic features. Such a difficulty has been recently overcome thanks to Deep Learning (DL)^26^. In particular, DL approaches based on customized or pre-trained convolutional neural networks (CNNs) have gained increasing attention^27^. Customised CNNs are networks built to accomplish a specific task requiring a high-cost and time-consuming training phase. Instead, pre-trained CNNs refer to transfer learning technique^27^: the networks have been previously trained (pre-trained CNNs) on a huge number (millions) of natural non-medical images to learn how to automatically extract features of different level of abstraction, low-level features, e.g., edge and dots, and high-level features, e.g., shapes and objects, from a raw image. Since the knowledge that the networks have acquired during training can be transferred and applied on never unseen images across diverse research fields (transfer learning), also including MRI data analysis, they are more generalizable and much less expensive with respect to the computational burden.

In general, both customized and pre-trained CNNs have been already successfully applied on medical imaging to detect^28^ or classify^29^ breast tumor masses. These networks have seen usage in the NAC framework for the prediction of pCR by taking in input all the possible DCE-MRI scans acquired during the course of the chemotherapy^30^ or only the exams prior to treatment^31–33^. In the latter case, the prediction of pCR is based on characteristics of the tumor at the time of initial diagnosis neglecting some features that can be related to the primary or overall effect of the therapy. Only in recent times, a customized CNN has been developed demonstrating how the early-treatment exam plays a key role to predict if the NAC treatment can be pursued or not depending on whether a patient is an early responder or not^34^. However, to the best of our knowledge, there is a lack of research works based on transfer learning to give an early evaluation of the effectiveness of NAC from its early stage by predicting pCR.

In this work, we exploited a transfer learning approach based on a pre-trained CNN that takes in input pre-treatment and early-treatment MRI exams from I-SPY1 TRIAL public database to evaluate “early on” the efficacy of NAC before the end of the therapy itself. Such a proposal represents the first effort towards the designing of a completely automatized support tool to better guide the treatment planning. Indeed, in accordance with the probability of reaching pCR for an individual patient, the medical figures can decide to pursue or change a specific treatment pathway.

## Results

### Data description and statistical analysis results

**Figure 1:**
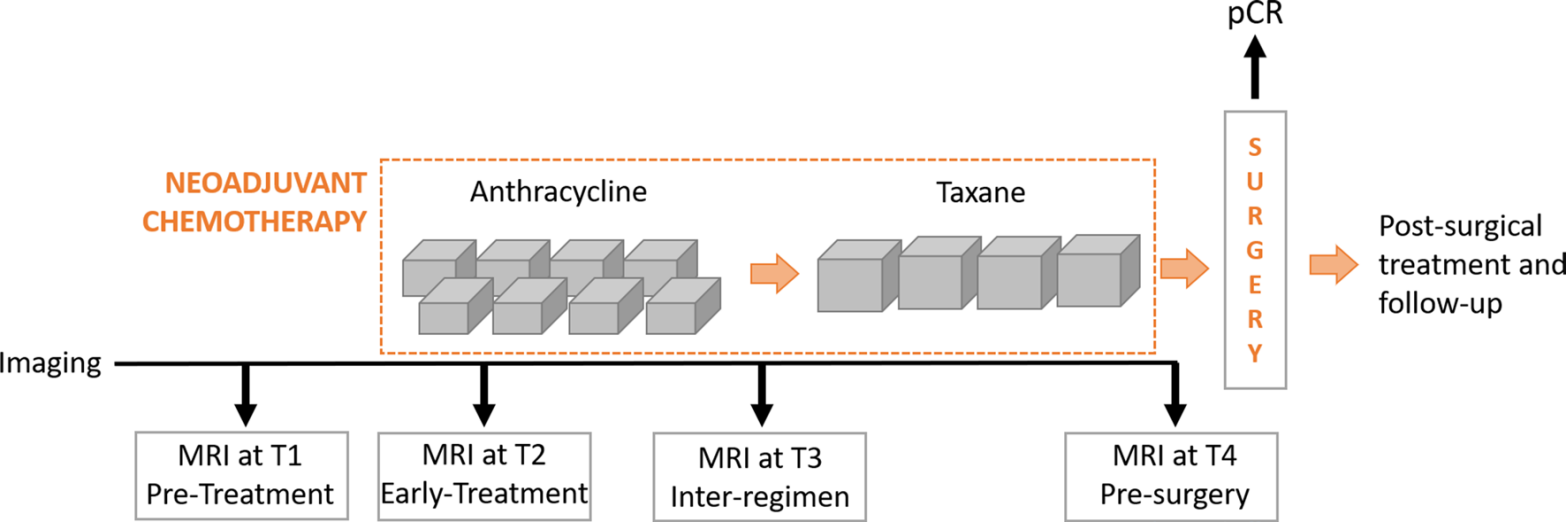
Chart of the MRI acquisitions for patients of the I-SPY1 TRIAL dataset undergoing neoadjuvant chemotherapy. Pre-treatment and early-treatment exams (MRI at T1 and MRI at T2, respectively) were analysed for the prediction of pathological Complete Response (pCR). The final treatment evaluation was performed at the end of chemotherapy and after the surgery by pCR.

Images used for the purpose of the presented study refer to a set of DCE-MRIs from the multi-site Investigation of Serial Studies to Predict Your Therapeutic Response with Imaging and molecular Analysis (I-SPY1 TRIAL)^7,35,36^ public dataset, which contains cases of 230 women enrolled between 2002 and 2006 with breast tumors of at least 3 cm in size, who received NAC with an anthracycline-cyclophosphamide (AC) regimen alone or followed by taxane. The dataset is available online on The Cancer Imaging Archive^37^. Each patient underwent a maximum of four MRI examinations (see Fig. 1): at around four weeks prior to treatment (MRI at T1, pre-treatment); at least two weeks after the first AC cycle and before the second AC cycle (MRI at T2, early-treatment); at the end of the AC cycle and the start of taxane treatment if taxane was administered (MRI at T3, inter-regimen); at the end of the entire chemotherapy cycle and prior to surgery (MRI at T4, pre-surgery). At each timepoint, three images were acquired using 1.5 T field-strength MR imaging systems^7^: a single pre-contrast image and two images corresponding to approximately 2 ½ minutes and 7 ½ minutes post contrast injection, respectively.

We formulated a binary classification task to distinguish responder patients (whose class was indicated as pCR) from non-responder patients (whose class was indicated as non-pCR). With the aim to provide an estimation of the NAC effect even since its first cycle in terms of pCR prediction, the first post-contrast MRI at T2 exams alone or combined with the first post-contrast MRI at T1 exams were analysed. The prediction of pCR by exploiting only the MRI at T1 exams was also evaluated.

In accordance with image availability (both MRI at T1 and MRI at T2 exams), 134 cases of study were identified. Each patient was finally represented by two Regions Of Interest (ROIs), one from MRI at T1 and one from MRI at T2 (see “Methods”). For each ROI, the number of extracted features by the pre-trained CNN AlexNET^38^ was 43,264 in total. Two subsets of patients were then taken into account. The first subset of patients, called fine-tuning dataset, involved 108 patients, out of which 30 patients achieved pCR and 78 did not. This subset was used to find an optimal subset of CNN-extracted features, as small as possible, that could be valid for any patient (see “Methods”). The second subset of patients (7 pCR; 19 non-pCR) was composed by patients not involved in the feature selection procedure and was thus used as independent test to validate the model. The division of patients in the two sets is explained in the Methods section.

In Table 1, the spitting of the 134 patients as responders (pCR, 37) and non-responders (non-pCR, 97) and their relative clinical information are summarized. The rates of responder patients (pCR) are significatively higher in Triple Negative and HER2+ subtypes, namely, 38% (14/37) and 43% (16/37) respectively, than in luminal subtype corresponding to 16% (6/37). Performing an association test between pCR to therapy and each clinical factor is essential to understand whether there is a relationship between the two variables or not. As a result, a significant association of the pCR with ER, PgR, HER2 and subtype (*p-*value Chi Square Test < 0.05) was found (see “Methods”). In our further analysis, only these last clinical variables were integrated to the CNN-extracted features.

**Table 1:** Clinical details about the patients involved in the study.

	pCR	non-pCR
**Number** (n = 134)	37 (30/7)	97 (78/19)
**Age** (years)	46.88 ± 8.77 (45.97 ± 8.40/50.81 ± 9.94)	48.84 ± 9.03 (49.04 ± 9.31/48.01 ± 7.97)
**Race**		
Caucasian	29 (22/7)	73 (56/17)
African American	3 (3/0)	18 (17/1)
Asian	2 (2/0)	5 (4/1)
Native Hawaiian/Pacific Islander	1 (1/0)	0
Multiple race	1 (1/0)	0
Not Identified	1 (1/0)	1 (1/0)
**Molecular subtype**		
HER2+	16 (12/4)	22 (19/3)
Luminal	6 (6/0)	51 (39/12)
Triple Negative	14 (11/3)	23 (19/4)

In the brackets the division of the patients between the fine-tuning dataset (first number) and the independent test (second number) is specified.

### Early evaluation of NAC efficacy through pCR prediction

**Figure 2:**
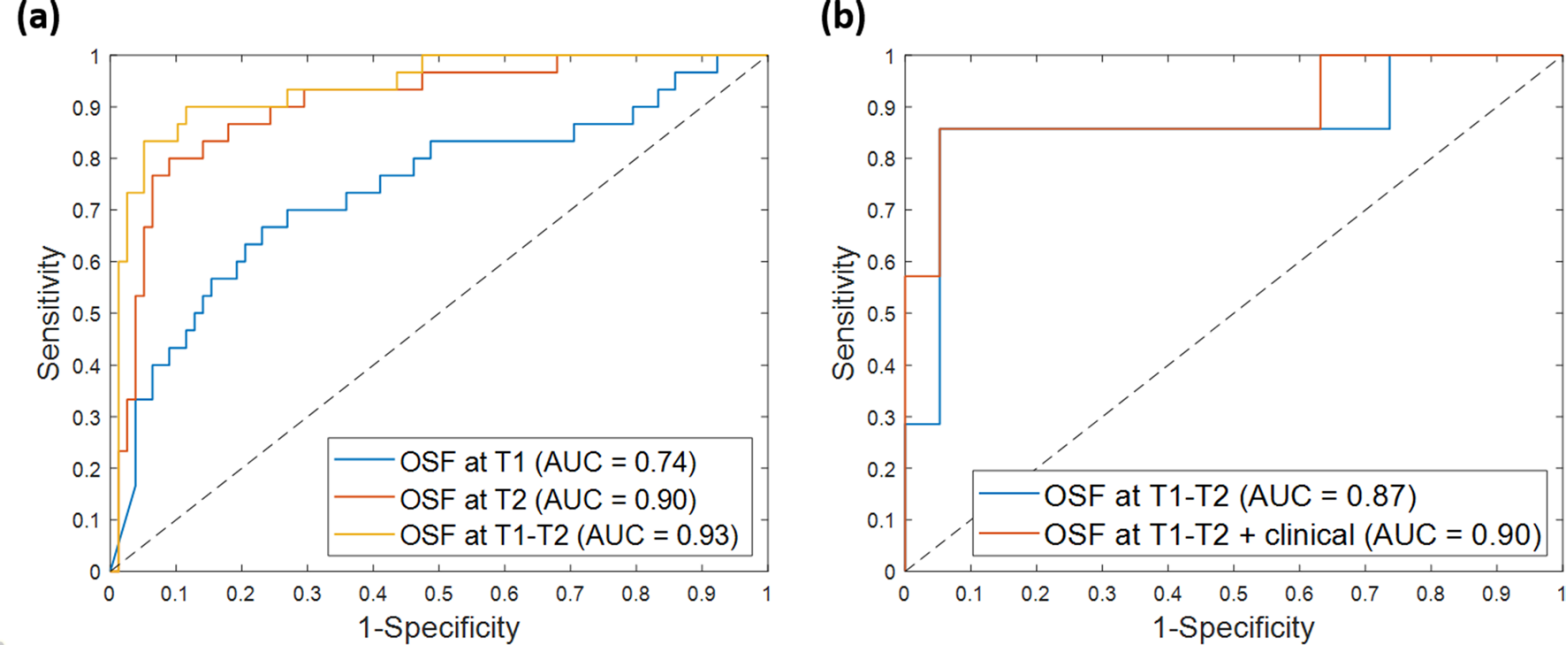
ROC curves for pCR prediction models. (**a**) ROC curves related to the model with the Optimal Subset of Features (OSF) at timepoints T1 and T2 separately or in combination and evaluated on the fine-tuning dataset. (**b**) ROC curves related to the best model (OSF at T1-T2) and the best model with clinical variables (OSF at T1-T2 + clinical) and evaluated on the independent test. (**a**, **b**) The AUC values are also highlighted.

The optimal subsets of selected features obtained through the implementation of *Step 3* (see “Methods”) on the fine-tuning dataset were referred as OSF at T1 for MRI at T1 exams alone, as OSF at T2 for MRI at T2 exams alone, and as OSF at T1-T2 for MRI at T1 and MRI at T2 exams combined, respectively. The number of these sets of features was 5 and 15 for OSF at T1 and OSF at T2, respectively. Therefore, 20 features were comprised in OSF at T1-T2. Table 2 summarizes the results on the fine-tuning dataset by designing the model with optimal features extracted from the exams at T1 or T2 separately or in combination. When the model included OSF at T1 alone, the classifier moderately distinguished responder from non-responder patients (accuracy of 74.1%, sensitivity of 66.7% and specificity of 76.9%). A higher performance was achieved using OSF at T2: accuracy of 88.0%, sensitivity of 81.0% and specificity of 91.0%. The best performance was obtained by combining the optimal tumor features related to the initial diagnosis and the early-treatment exam (OFS at T1-T2): an accuracy of 88.9%, a sensitivity of 90.0% and a specificity of 88.5% were returned. In the latter case, also the AUC was the best value achieved: a value of 0.93 was reached with OSF at T1-T2 with respect to 0.90 and 0.74 returned with OSF at T2 and OSF at T1, respectively. In Fig. 2a, the AUC values as well as the corresponding Receiver Operating Characteristic (ROC) curves are drawn.

**Table 2:** Summary of the performances of the pCR prediction models in terms of accuracy, sensitivity, and specificity on the fine-tuning dataset and the independent test.

Set	Model	N. features	Accuracy (%)	Sensitivity (%)	Specificity (%)
**Fine-tuning dataset**	**OSF at T1**	5	74.1	66.7	76.9
	**OSF at T2**	15	88.0	80.0	91.0
	**OSF at T1-T2**	5 + 15	88.9	90.0	88.5
	Clinical	4	73.4	34.5	88.3
	**OSF at T1 + clinical**	5 + 4	75.9	70.0	78.2
	**OSF at T2 + clinical**	15 + 4	89.5	72.4	96.1
	**OSF at T1-T2 + clinical**	5 + 15 + 4	91.4	79.3	96.1
**Independent test**	**Clinical**	4	69.2	42.9	78.9
	**OSF at T1-T2**	15 + 4	92.3	85.7	94.7
	**OSF at T1-T2 + clinical**	5 + 15 + 4	92.3	85.7	94.7

The number of features composing each model is also reported.

We also investigated the performance of the best model (with features belonging to OSF at T1-T2) when the patients of the fine-tuning dataset were divided according to the molecular subtypes of breast cancer:

1. luminal encompassing 45 patients (6 pCR; 39 non-pCR);
2. HER2+ involving 31 patients (12 pCR; 19 non-pCR);
3. Triple Negative including 30 patients (11 pCR; 19 non-pCR).

The sum of patients belonging to the three subtypes was not 108 but 106 because for two patients the HER2+ variable was not specified. The model perfectly discriminated responders from non-responders from the luminal subtype. A good performance was reached on the HER2+ subtype with an accuracy of 83.9%, a sensitivity of 91.7%, a specificity of 78.9%. Finally, the model returned an accuracy of 90.0%, a sensitivity of 81.8%, a specificity of 94.7% for the Triple Negative subtype.

As further step of our analysis, we probed the performance of the proposed model on the fine-tuning dataset when the four clinical variables that showed an association with pCR were integrated: ER, PgR, HER2, subtype (see the previous paragraph). In Table 2, the performances achieved by an SVM classifier designed either with the only clinical variables (indicated as clinical) or with the clinical variables added to the optimal features related to timepoints T1 and T2 separately or in combination (indicated as OSF at T1 + clinical, OSF at T2 + clinical and OSF at T1-T2 + clinical, respectively) are also represented.

When joined together to design an SVM classifier, the clinical variables alone showed an accuracy of 73.4%, a sensitivity of 34.5%, a specificity of 88.3% and an AUC value of 0.42. However, the addition of clinical variables boosted the performance of the proposed model when only the optimal features related to timepoint T1 were considered (OSF at T1 + clinical) achieving an accuracy of 75.9%, a sensitivity of 70.0% and a specificity of 78.2%. The AUC value passed from 0.74 to 0.76. Moreover, when the clinical variables were added to the optimal features from early-treatment exams alone (OSF at T2 + clinical) or to features from both pre-treatment and early treatment exams (OSF at T1-T2 + clinical), an improvement of specificity (96.1%) and accuracy (89.5% and 91.4%, respectively) was obtained. AUC values of 0.91 and 0.93 were returned, respectively.

Finally, the performance of the best model with CNN-extracted features (OSF at T1-T2) was evaluated on the independent test (see Table 2). An accuracy of 92.3%, a sensitivity of 85.7% and a specificity of 94.7% were achieved. As well as for the fine-tuning dataset, the clinical variables alone were not able to effectively discriminate responder from non-responder patients of the independent test (clinical). Anyway, combining the four clinical information with the optimal features from both pre-treatment and early-treatment exams (OSF at T1-T2 + clinical) led to maintain the same values of accuracy, sensitivity, and specificity. The AUC value increased from 0.87 to 0.90. The corresponding ROC curves are depicted in Fig. 2b.

### Visual cue of convolutional features

**Figure 3:**
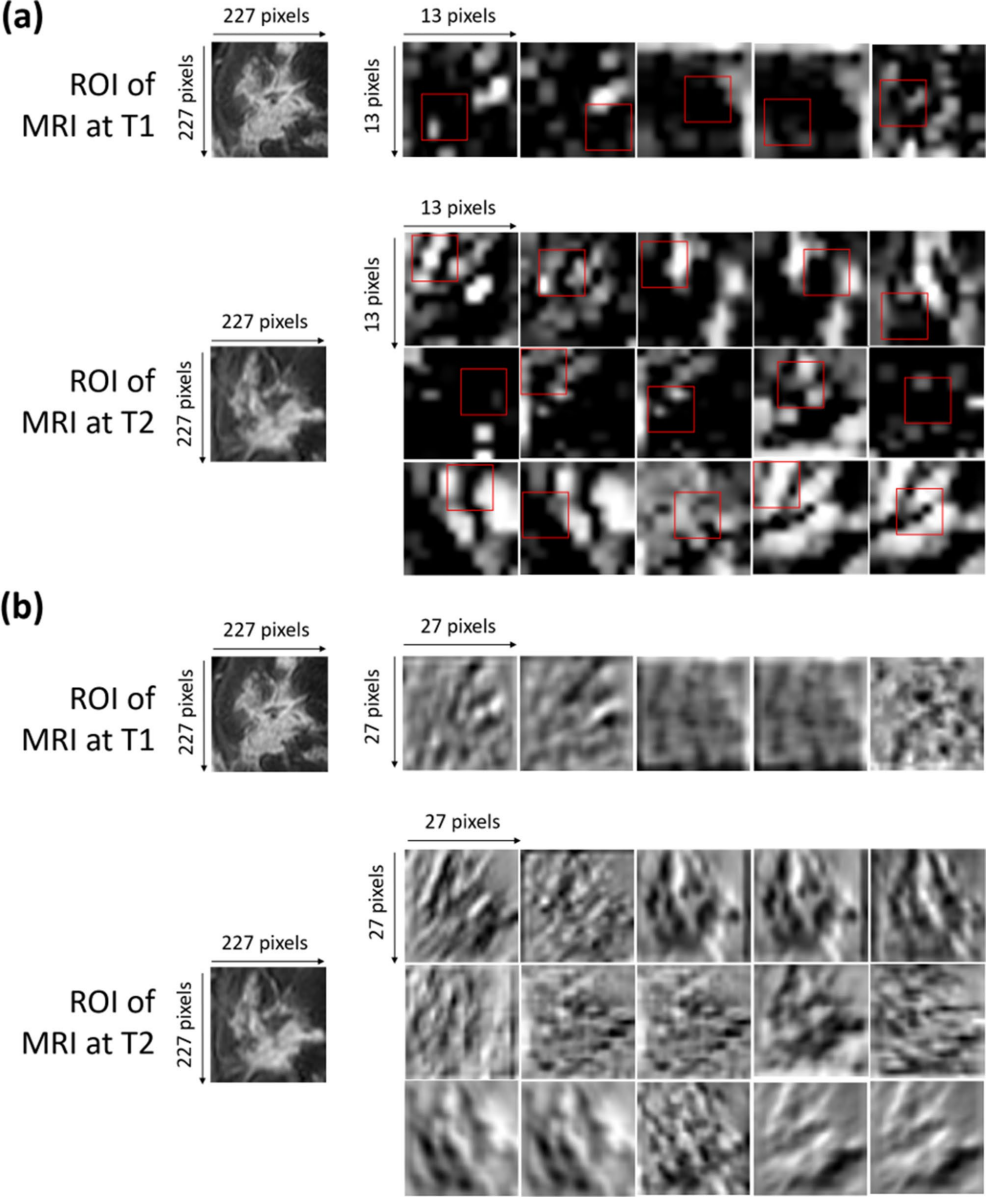
(**a**) Activation maps of the ROIs of both MRI at T1 and MRI at T2 from which the selected optimal features were extracted. The red squares outline with more precision the area of belonging of such features. Each ROI has dimensions of 227 $\times$ 227 pixels. Each activation map has original dimensions of 13 $\times$ 13 pixels and has been resized to the dimensions of the corresponding ROIs for a better visualization. (**b**) Convolutional maps related to the activation maps of the ROIs of both MRI at T1 and MRI at T2 from which the selected optimal features were extracted. Each ROI has dimensions of 227 $\times$ 227 pixels. Each convolutional map has original dimensions of 27 $\times$ 27 pixels and has been resized to the dimensions of the corresponding ROI for a better visualization.

The convolutional features, which were here identified as part of the OSF at T1-T2, were extracted by an inner layer (*pool 2*) of the given pre-trained CNN architecture. A direct understanding of which image portion these features represent is made no trivial due to the complex non-linear operations computed within the network architecture. However, a visual cue of the convolutional features selected as optimal ones may be very informative and interesting for a deeper understanding of the CNN functioning. To do this, for two different ROIs, one from MRI at T1 exam and one from MRI at T2 exam, we extracted the so-called activation maps to which the selected optimal features belong (Fig. 3a). The red squares in Fig. 3a outline with more precision the area of belonging of the selected features. The corresponding convolutional maps are also highlighted to visualize the operation that has been applied within the network starting from the original ROIs after a primary convolution (Fig. 3b). Basically, each convolutional map shows what kind of lines or details are extrapolated from the corresponding original image. Since the extracted features are low-level characteristics of the images, they refer to edges, lines or points of the image. The 15 features selected from MRI at T2 exams seem to cover all the tumor mass zones. The convolutional features extracted from MRI at T1 exams seem to principally refer to edges or peripheral zones of the tumor mass.

## Discussion

The role of MR imaging has been demonstrated to be crucial in detecting the pathological complete response to neoadjuvant chemotherapy^7–9^ also revealing a greater detection accuracy than other diagnostics techniques, such as mammography^10^. With the emergence and the spread of the modern concept of personalized medicine, an early prediction of pathological complete response for breast cancer patients undergoing neoadjuvant chemotherapy through the analysis of MRI examinations has become a topic of great interest in the state of the art. An accurate prediction of tumor response allows indeed the medical staff to select or design a patient-centric approach consisting in an optimal treatment and care pathway for each individual patient. Some research works have been addressed to an early evaluation of NACT efficacy, i.e., from its early stages, by means of the prediction of the final pCR^1,7,14,20–23^. Among these works, Hylthon et al.^7^ analysed MRI exams from 216 patients of the I-SPY1 TRIAL database reporting that MR imaging findings are stronger predictors of pCR to NAC than clinical assessment. In multivariate analysis using a random-effects logistic regression model, an AUC value of 0.73 was achieved by combining MR tumor measurements (changes in longest dimension, volume, and SER) from early-treatment exams and clinical size. Despite the promising results, these studies require an expert interpretation and a manual feature extraction that can be limited by human bias: analysts can project features according to their knowledge limited by human imagination thus not devising all possible useful features for the task under study. More recently, a paradigm shift based on deep learning have been proposed to analyse medical images among which MRI exams. The innovative idea is to use customised or pre-trained CNNs to automatically extract features from images without human intervention and bias^26^.

So far, there have been several attempts to develop approaches based on customised CNNs with the goal to predict pCR to NAC for breast patients using the only pre-treatment MRI exams from I-SPY1 TRIAL public database. Liu et al.^32^ developed a customised CNN exploiting first post-contrast pre-treatment MRI examinations from 131 patients (40 pCR; 91 non-pCR): a mean AUC value of 0.72 was reached. Similarly, Ravichandran et al.^33^ designed a customised CNN utilizing pre-contrast and post-contrast pre-treatment MRI scans in isolation or in conjunction. The network was trained with images of 133 patients and tested on 33 patients. When only the post-contrast MRI exams were considered, an AUC value of 0.70 was returned. These models based on customised networks measured features of tumor masses with the aim to answer if it is possible to define *a-priori*, i.e., before the starting of NAC, if the therapy can succeed or not. However, the analysis of early-treatment exams can be essential to determine if continuing or changing the specific a-priori chosen NAC regimen depending on whether the patient is an early responder or not. In the recent past, El Adoui et al.^34^ proposed a customised CNN based on pre and post- first chemotherapy MRI images of a cohort of 42 patients, who underwent a NAC treatment consisting of three cycles of chemotherapies followed by docetaxel drug given every 3 weeks for 3 to 4 cycles or paclitaxel drug weekly for 3 months. Using a validation set of 14 cases, the model reached an AUC value of 0.91.

Within this constantly evolving scenario, in the current work, we proposed a transfer learning approach to predict the efficacy of NAC from its earliest stage in terms of pCR. As far as we know, this task has been not extensively investigated through transfer learning techniques. We investigated the role of the convolutional features extracted from pre-treatment and early-treatment MRI exams of breast patients from I-SPY1 TRIAL database in predicting pCR. Promising results were achieved by testing the approach after the application of a feature selection method on the so-called fine-tuning dataset to identify the most stable features. The ability of these features to discriminate the responder patients from the non-responder ones has been also performed across the three molecular subtypes of breast cancer. Moreover, the robustness of the model has been evaluated on an independent test whose patients were not involved in the feature selection process. By combining the optimal features extracted from both pre-treatment and early-treatment exams with some clinical features, i.e., ER, PgR, HER2 and molecular subtype, an accuracy of 91.4% and 92.3%, and an AUC value of 0.93 and 0.90, were returned on the fine-tuning dataset and the independent test, respectively. The numerical results demonstrate that a reliable evaluation of NAC efficacy can be obtained even since its earliest stage. The analysis of both pre-treatment and early-treatment exams can give us complementary information about the disease: while the characteristics of the tumor masses at the time of initial diagnosis can afford an intuition of tumor response to treatment, the tumor features identified after the first cycle can measure the primary effect of the therapy. Overall, the low-level CNN-extracted features reveal to effectively provide an early prediction of NAC effect in terms of pCR.

With respect to the pCR predictive models mentioned before, we did not refer to a customized network but to a pre-trained and more basic network that exploited only low-level features. Conversely to pre-trained networks, customised CNNs are networks built to accomplish a specific task. Training and test data are drawn from the same distribution or belong to the same application field. They require a training phase that involves a large number of data and usually lasts from hours to days. However, sustaining these high computational burden and processing capabilities is not always feasible. In contrast, using a pre-trained network allows, on the one side, to reduce time-consuming because they do not require any training and, on the other side, to improve generalizability across several domains of application. Since no technical expertise is required in the extraction of meaningful features from images, the proposed model is more prone to an easy interpretation and utilization. The main purpose of the work is proposing a first effort towards the designing of a completely automatized support tool that could help medical figures to evaluate “early on” the efficacy of NAC before the end of the therapy itself, in order to better guide their therapeutic choices in accordance with the probability of reaching pCR. The main factors favouring the choice of NAC are a high ratio between the tumor volume and the breast volume, the presence of lymph node metastases, the young age of the patient and some histological parameters including the high tumor grading and the presence of specific molecular subtypes such as Triple Negative and HER2+ tumors^2^. Therefore, the proposed support tool could be helpful to drive any changes to the therapy in progress, i.e., dose-dense neoadjuvant chemotherapy^39^, introduction of new chemotherapy drugs or combination of chemotherapy and other drugs, such as trastuzumab^40^.

Manifold are the paths of possible future extensions of this work. The relatively small size of the dataset employed for our analysis represent one of the limitations of this study. The generalizability and flexibility of the proposed method can be made stronger thanks to the validation of the method on larger datasets also including patients registered from our Institute. Although we have explored the role of the clinical variables if they considered alone or combined with the CNN-extracted features, other kinds of clinical variables, such as gene profiling, could be involved in the analysis. Several technical aspects can be probed in deeper. First, the performances of the model can be discussed at varying the feature selection method: several filter selection methods with different cut-off values to define the statistically significative difference and even more sophisticated feature selection techniques, such as wrapper and embedded methods, can be performed. Second, more complex features to extract from the final layers of the network could be also added or the temporal relationship among features related to subsequent exams could be investigated. Alongside all these developments, the proposed tool can be extended to evaluate the efficacy of multiple neoadjuvant schemes early on. In this way, the probability of positively responding to therapy could be predicted and compared among the diverse NAC scenarios referred to the ASCO guidelines^40^. The medical experts could be able to prefer a tailored therapy pathway associated with the highest probability of positive response to therapy.

## Methods

### Data pre-processing

Tumor segmentations related to the fine-tuning dataset were part of the online dataset and were automatically generated by thresholding semi-quantitative pharmacokinetic parameters, peak enhancement, and signal enhancement ratio. Then, we selected another set of patients, i.e., the independent test, for which both MRI at T1 and MRI at T2 exams were available without tumor segmentations. In this case, starting from the area containing the lesion that was identified by our expert breast imaging radiologist with over 20 years of experience, tumor segmentations have been performed by the same procedure used for the images of the fine-tuning dataset^35^. With the aim to analyse not only the area containing the lesion but also the immediately surrounding peritumoral area, for the images of both sets of patients, a final ROI of size equals to the Largest Diameter (LD) of the tumor, whose value was reported in a clinical report available as part of the online database, was centred around the centre of the tumor mass from the slice of the MRI scan with the largest tumor area. ROI identification was executed on the first post-contrast MRI at T1 and MRI at T2 scans separately. Each patient was finally represented by two ROIs, one from MRI at T1 and one from MRI at T2. All the ROIs were resized to patches of size of 227 $\times$ 227 pixels in order to be given in input to the network used for feature extraction, since the network requires images of such size as input.

Finally, we have preferred to make a first validation of the model using a LOO cross-validation procedure on the images for which the area containing the lesion was already identified (fine-tuning dataset). Then, we have evaluated the performance of the entire analysis workflow (from semi-automatic segmentation to classification) on images that did not have an indication of the area containing the lesion (independent test). In this way, we were able to achieve an evaluation of the performances of the model close to those that the model could reach in the real clinical application.

### Pipeline of the proposed transfer learning approach

**Figure 4:**
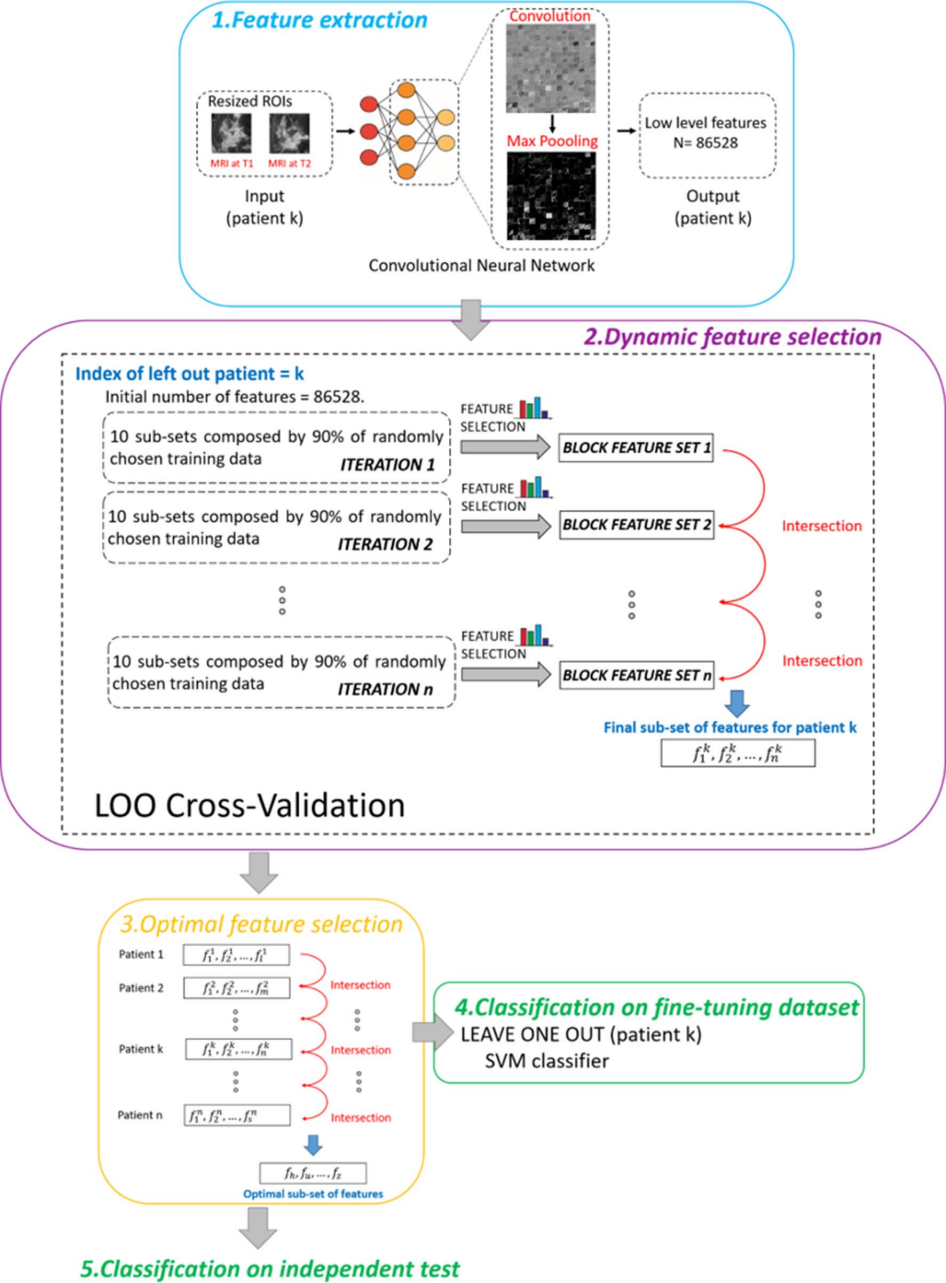
Workflow of the proposed transfer learning method encompassing five steps: 1. Feature extraction, 2. Dynamic feature selection, 3. Optimal feature selection, 4. Classification on the fine-tuning dataset, 5. Classification on the independent test.

We proposed a transfer learning approach for an early evaluation of NAC efficacy by predicting the final pCR. The method was composed of a feature extractor, such as a convolutional neural network (CNN), called AlexNET^38^, that was pre-trained on non-medical images, and a standard classifier, such as Support Vector Machine (SVM)^41^, that predicted if a patient achieved pCR or did not. The pipeline of the proposed method is depicted in Fig. 4 and explained below step by step. All the steps were performed by using the MATLAB R2019a (MathWorks, Inc., Natick, MA, USA) software.

- *Step 1*. Feature extraction

Inspired by the latest success of the so-called *transfer learning* technique across disparate fields of application^29,42,43^, we used a high-performing pre-trained DL architecture based on CNNs, called AlexNET, as feature extractor^38^. Such a network has been previously trained on millions of images belonging to thousands of categories. The knowledge learned by the network during the training phase was here transferred on our images. So, we exploited the transfer learning technique to extract features from the MR images and utilize them to fulfil the classification task, pCR *vs* non-pCR.

In the present work, we extracted features from the *pool2 layer* of the network architecture which corresponds to the second pooling layer (Max Pooling in phase 1 of Fig. 4) after the second convolutional layer of the network (Convolution in phase 1 of Fig. 4). The *pool2 layer* had an output with dimensions of 13 $\times$ 13 $\times$ 256 that was flattening to a single 43,264-length vector. As consequence, for each ROI, the number of extracted features was 43,264 in total. Since the *pool2 layer* is one of the initial layers of the network, the corresponding extracted features are low-level features, namely, representations of local details of an image, such as edges, dots, and curves^44^. Within the network architecture, low-level features are combined to build high-level features, that refer to global cues of an image, such as shapes or entire objects. The latter features are learned from later layers of the network from which they can be extracted. We considered here low-level features in order to dissect information deriving from all the local structures of the images that could be obscured instead by considering only global information. In addition, we extracted the features not directly from a convolution layer that returns the feature maps but after the application of pooling that, as well-known in deep learning theory^45^, makes features invariant to truncation, occlusion, and translation. Finally, the usage of a transfer learning approach was here preferred to a customized network because it provides some benefits especially when, as in our case, a relatively small amount of data is available. When a pre-trained network is used as a feature extractor only, no training phase is required. As a consequence, a drastic reduction of the computational time occurs with respect to use customized networks for which the training phase is indispensable and time-consuming (from hours to days). Not less relevant, especially for datasets no large enough, customized neural networks are more prone to the risk of overfitting. Conversely, the possibility to use a pre-trained net allows high generalizability of the results.

- *Step 2*. Dynamic feature selection

A dynamic feature selection procedure was performed on the fine-tuning dataset by considering either the features extracted from ROIs of MRI at T1 or MRI at T2 exams in isolation (43,264 features per patient, respectively) or the features extracted from ROIs of MRI at T1 and MRI at T2 exams in conjunction (86,528 features per patient). This feature selection was executed according to a Leave-One-patient-Out (LOO) cross-validation procedure for which one patient was leaved out at each step. We addressed such a technique as *dynamic* feature selection because it selected a diverse set of features in correspondence of each patient leaved out. At each step of LOO cross-validation, an iterative procedure that consisted of stacking 10 subsets with 90% of randomly chosen training samples (see phase 2 in Fig. 4) was applied. At each iteration, for each of the training subsets, we reduced the high dimensionality of the dataset by means of a well-known feature selection method that falls in the category of filter methods. We used the non-parametric Wilcoxon-Mann–Whitney test^46^ that compared the medians of the distributions related to the two classes (pCR *vs* non-pCR) and verified whether they were equal. *p*-value equals to 0.001 was set as cut-off to indicate a statistically significative difference between pairs of analysed distributions.

The features selected for each subset were pulled together in a single set. At one iteration corresponded one subset of features (see phase 2 in Fig. 4). The subsets of features obtained from following iterations were intersected among each other resulting in a single feature set as outcome of each LOO step. The rationale under the iterative process was to iteratively reduce the number of features in order to finally obtain a set of more “stable” features. Those features could be considered the ones that were always preferred by the feature selection algorithm and thus less susceptible to variations of training samples^47,48^, which is typical when high-dimensional datasets are treated. The total number of iterations (*n*) was empirically estimated as *n* = 20.

- *Step 3*. Optimal feature selection

To address the problem of feature stability on high-dimensional dataset, in *Step 2* we figured out a strategy to obtain subsets of more stable features. However, each patient was characterized by a feature subset that differed from other patients. With this new step, we wanted to identify a single subset of features that was meaningful for all the patients of the fine-tuning dataset. In practice, an Optimal Subset of Features (referred as OSF) was obtained by intersecting all the subsets of features (one for each patient) that were outcome of the *Step 2* either when only the features extracted from ROIs of MRI at T1 or MRI at T2 were considered or when features extracted from ROIs of both MRI at T1 and MRI at T2 were involved in the process. The optimal subsets of selected features were referred as OSF at T1, OSF at T2 and OSF at T1-T2, respectively.

- *Step 4*. Classification on the fine-tuning dataset

The power of the optimal selected features in discriminating responder from non-responder patients was assessed by defining three SVM classifiers in correspondence to OSF at T1, OSF at T2 and OSF at T1-T2, respectively. Their performances evaluation was executed by a leave-one-patient-out cross-validation procedure. All the classifiers were then separately augmented by some clinical variables. Moreover, the best model (composed by features belonging to OSF at T1-T2, see “Results”) was also evaluated across the three molecular subtypes of breast cancer.

- *Step 5*. Classification on independent test

The best model on the fine-tuning dataset (with features belonging to OSF at T1-T2, see “Results”) was finally evaluated on the independent test whose patients were not involved in the feature selection process. More specifically, an SVM classifier was constructed using the fine-tuning dataset as training set and exploiting features belonging to OSF at T1-T2. The addition of some clinical variables was also investigated.

### Statistical analysis and performance evaluation

The clinical variables included as part of the online information of the public database were age, race, ER, PgR, HER2, and the specification of what molecular subtype among luminal, HER2+, Triple Negative the patient belonged (named as subtype). In order to evaluate the association between each clinical feature and pCR, we used Mann–Whitney test for the age feature, whereas we used Chi-square test for all the other features measured on an ordinal scale. A result was considered statistically significant when the *p*-value was less than 0.05. The classification performances (pCR *vs* non-pCR) of the all the models mentioned in *Steps 4–5* of the previous paragraph were evaluated in terms of the Area Under the receiver operating Curve (AUC) and, once the optimal threshold was identified by Youden’s index on ROC curves^49^, standard metrics, such as accuracy, sensitivity and specificity were also computed.

### Ethics approval and consent to participate

The study was conducted according to the guidelines of the Declaration of Helsinki, and approved by the Scientific Board of Istituto Tumori ‘Giovanni Paolo II’ (Bari, Italy)—Prot. 6629/21.

### Consent for publication

Images used for the purpose of the presented study refer to a set of DCE-MRIs from the multi-site Investigation of Serial Studies to Predict Your Therapeutic Response with Imaging and molecular Analysis (I-SPY1 TRIAL) public dataset.

## Data Availability

The dataset analyzed during the current study is available in the Cancer Imaging Archive, https://wiki.cancerimagingarchive.net/display/Public/ISPY1.

## References

[1] Eun NL (2020). Texture analysis with 3.0-T MRI for association of response to neoadjuvant chemotherapy in breast cancer. Radiology.

[2] Cain H (2017). Neoadjuvant therapy in early breast cancer: treatment considerations and common debates in practice. Clin. Oncol..

[3] Rustin GJS (2004). Re: New guidelines to evaluate the response to treatment in solid tumors (Ovarian Cancer) [2]. J. Natl. Cancer Inst..

[4] Eisenhauer EA (2009). New response evaluation criteria in solid tumours: Revised RECIST guideline (version 1.1). Eur. J. Cancer.

[5] Caudle AS (2011). Impact of progression during neoadjuvant chemotherapy on surgical management of breast cancer. Ann. Surg. Oncol..

[6] Cho JH (2013). Oncologic safety of breast-conserving surgery compared to mastectomy in patients receiving neoadjuvant chemotherapy for locally advanced breast cancer. J. Surg. Oncol..

[7] Hylton NM (2012). Locally advanced breast cancer: MR imaging for prediction of response to neoadjuvant chemotherapy - Results from ACRIN 6657/I-SPY TRIAL. Radiology.

[8] Loo CE (2011). Magnetic resonance imaging response monitoring of breast cancer during neoadjuvant chemotherapy: Relevance of Breast Cancer Subtype. J. Clin. Oncol..

[9] Marinovich ML (2012). Early prediction of pathologic response to neoadjuvant therapy in breast cancer: Systematic review of the accuracy of MRI. Breast.

[10] Scheel JR (2018). MRI, clinical examination, and mammography for preoperative assessment of residual disease and pathologic complete response after neoadjuvant chemotherapy for breast cancer: ACRIN 6657 trial. Am. J. Roentgenol..

[11] Su, M. Y. L. Breast cancer: Early prediction of response to neoadjuvant chemotherapy using parametric response maps for MR imaging: Cho N, im S-A, Park I-A, et al (Seoul Natl Univ College of Medicine, Republic of Korea) Radiology 272:385–396, 2014. *Breast Dis.* **26**, 134–137 (2015). 10.1148/radiol.14131332 24738612

[12] Li X (2015). Multiparametric magnetic resonance imaging for predicting pathological response after the first cycle of neoadjuvant chemotherapy in breast cancer. Invest. Radiol..

[13] Fangberget A (2011). Neoadjuvant chemotherapy in breast cancer-response evaluation and prediction of response to treatment using dynamic contrast-enhanced and diffusion-weighted MR imaging. Eur. Radiol..

[14] Sharma U, Danishad KKA, Seenu V, Jagannathan NR (2009). Longitudinal study of the assessment by MRI and diffusion-weighted imaging of tumor response in patients with locally advanced breast cancer undergoing neoadjuvant chemotherapy. NMR Biomed..

[15] Beresford MJ (2006). Inter- and intraobserver variability in the evaluation of dynamic breast cancer MRI. J. Magn. Reson. Imaging.

[16] Bellotti R (2004). The MAGIC-5 project: Medical applications on a grid infrastructure connection. IEEE Nucl. Sci. Symp. Conf. Rec..

[17] Losurdo L (2019). Radiomics analysis on contrast-enhanced spectral mammography images for breast cancer diagnosis: A pilot study. Entropy.

[18] Fanizzi A (2019). Ensemble discretewavelet transform and gray-level co-occurrence matrix for microcalcification cluster classification in digital mammography. Appl. Sci..

[19] Fanizzi A, Basile TMA, Losurdo L, Amoroso N, Bellotti R, Bottigli U, Dentamaro R, Didonna V, Fausto A, La Forgia D (2017). Hough transform for clustered microcalcifications detection in full-field digital mammograms. Appl. Digit. Image Process. XL.

[20] Braman NM (2017). Intratumoral and peritumoral radiomics for the pretreatment prediction of pathological complete response to neoadjuvant chemotherapy based on breast DCE-MRI. Breast Cancer Res..

[21] Jahani N (2019). Prediction of treatment response to neoadjuvant chemotherapy for breast cancer via early changes in tumor heterogeneity captured by DCE-MRI registration. Sci. Rep..

[22] Tahmassebi A (2019). Impact of machine learning with multiparametric magnetic resonance imaging of the breast for early prediction of response to neoadjuvant chemotherapy and survival outcomes in breast cancer patients. Invest. Radiol..

[23] Lo Gullo R, Eskreis-Winkler S, Morris EA, Pinker K (2020). Machine learning with multiparametric magnetic resonance imaging of the breast for early prediction of response to neoadjuvant chemotherapy. Breast.

[24] Arasu VA (2019). Population-based assessment of the association between magnetic resonance imaging background parenchymal enhancement and future primary breast cancer risk. J. Clin. Oncol..

[25] Forgia, D. La *et al.* Response predictivity to neoadjuvant therapies in breast cancer: A qualitative analysis of background parenchymal enhancement in DCE-MRI, *Journal of Personalized Medicine*, **11**, 256 (2021). 10.3390/jpm11040256 PMC8065517 33915842

[26] LeCun Y, Bengio Y, Hinton G (2015). Deep learning. Nature.

[27] Panigrahi S, Nanda A, Swarnkar T (2009). A survey on transfer learning. IEEE Trans. Knowl. Data Eng..

[28] Wang Z (2019). Breast cancer detection using extreme learning machine based on feature fusion with CNN deep features. IEEE Access.

[29] Yu SD, Liu LL, Wang ZY, Dai GZ, Xie YQ (2019). Transferring deep neural networks for the differentiation of mammographic breast lesions. Sci. China Technol. Sci..

[30] Huynh, B. Q., Antropova, N. & Giger, M. L. Comparison of breast DCE-MRI contrast time points for predicting response to neoadjuvant chemotherapy using deep convolutional neural network features with transfer learning. *Med. Imaging 2017 Comput. Diagnosis* **10134**, 101340U (2017).

[31] Ha R (2019). Prior to initiation of chemotherapy, can we predict breast tumor response? Deep learning convolutional neural networks approach using a breast MRI tumor dataset. J. Digit. Imaging.

[32] Liu MZ (2020). A novel CNN algorithm for pathological complete response prediction using an I-SPY TRIAL breast MRI database. Magn. Reson. Imaging.

[33] Ravichandran, K., Braman, N., Janowczyk, A. & Madabhushi, A. A deep learning classifier for prediction of pathological complete response to neoadjuvant chemotherapy from baseline breast DCE-MRI. 11 (2018). 10.1117/12.2294056

[34] El Adoui M, Drisis S, Benjelloun M (2020). Multi-input deep learning architecture for predicting breast tumor response to chemotherapy using quantitative MR images. Int. J. Comput. Assist. Radiol. Surg..

[35] Newitt D, Hylton N (2016). Multi-center breast DCE-MRI data and segmentations from patients in the I-SPY 1/ACRIN 6657 trials. Cancer Imaging Arch..

[36] Hylton NM (2016). Neoadjuvant chemotherapy for breast cancer: Functional tumor volume by MR imaging predicts recurrencefree survival-results from the ACRIN 6657/CALGB 150007 I-SPY 1 TRIAL. Radiology.

[37] Clark K (2013). The cancer imaging archive (TCIA): Maintaining and operating a public information repository. J. Digit. Imaging.

[38] Russakovsky O (2015). ImageNet Large Scale Visual Recognition Challenge. Int. J. Comput. Vis..

[39] Ding Y (2020). Does dose-dense neoadjuvant chemotherapy have clinically significant prognostic value in breast cancer?: A meta-analysis of 3,724 patients. PLoS ONE.

[40] Korde LA (2021). Neoadjuvant chemotherapy, endocrine therapy, and targeted therapy for breast cancer: ASCO guideline. J. Clin. Oncol..

[41] Burges CJ (1998). A Tutorial on Support Vector Machines for Pattern Recognition. Data Min. Knowl. Discov..

[42] Casti P (2019). Calibration of vision-based measurement of pain intensity with multiple expert observers. IEEE Trans. Instrum. Meas..

[43] Mencattini A (2020). Discovering the hidden messages within cell trajectories using a deep learning approach for in vitro evaluation of cancer drug treatments. Sci. Rep..

[44] Salakhutdinov R, Tenenbaum JB, Torralba A (2013). Learning with Hierarchical-Deep Models. IEEE Trans. Pattern Anal. Mach. Intell..

[45] Zheng, L., Zhao, Y., Wang, S., Wang, J. & Tian, Q. Good Practice in CNN Feature Transfer. arXiv Prepr. arXiv:1604.00133. (2016).

[46] Mann HB, Whitney DR (1947). On a test of whether one of two random variables is stochastically larger larger than the other. Ann. Math. Stat..

[47] Nogueira S, Sechidis K, Brown G (2018). On the stability of feature selection algorithms. J. Mach. Learn. Res..

[48] Kalousis A, Prados J, Hilario M (2007). Stability of feature selection algorithms: A study on high-dimensional spaces. Knowl. Inf. Syst..

[49] Youden WJ (1950). Index for rating diagnostic tests. Cancer.

